# Distribution of metals and metalloids in dried seaweeds and health risk to population in southeastern China

**DOI:** 10.1038/s41598-018-21732-z

**Published:** 2018-02-23

**Authors:** Qing Chen, Xiao-Dong Pan, Bai-Fen Huang, Jian-Long Han

**Affiliations:** Zhejiang Provincial Center for Disease Control and Prevention, Hangzhou, China

## Abstract

Concern about metals and metalloids, especially heavy metals in seaweeds has risen due to potential health risk. This study investigated the distribution of 10 metals and metalloids in 295 dried seaweeds (brown and red) and estimated the possible health risk via hazard index (HI). Elements in seaweeds can be sequenced in descending order by mean values: Al > Mn > As > Cu > Cr > Ni > Cd > Se > Pb > Hg. The levels of Cd, Cu, Mn and Ni in red seaweeds were significantly higher than those in brown seaweeds (*P* < 0.01). Correlation analysis showed contents of Ni-Cr (*r* = 0.59, *P* < 0.01) in seaweeds had moderate positive correlations. Seaweeds from different geographical origins had diverse element distribution. Risk assessment showed that HI at mean level was less than the threshold of 1. It indicates that for the general people there is low health risk to these elements by the intake of seaweeds. Furthermore, in terms of the confirmative toxicity of some metals, such as Cd, Pb and Hg, surveillance of metals in seaweeds should be performed continuously.

## Introduction

Seaweed is increasingly consumed as one of dietary foods due to their abundance of natural vitamins, minerals and plant-based protein. In 2014, the production of farmed seaweeds reached 27.3 million tonnes, and the main cultured seaweeds were carrageenan seaweeds (including *Kappaphycus alvarezii* and *Eucheuma spp*.) and Japanese kelp (*Saccharina/Laminaria japonica*)^1^. For pacific countries, especially developing countries, large amounts of seaweeds are not only foods, but also represent an important economic resource.

Recently, concern has been raised about possible heavy metals contamination in seaweeds. Anthropogenic sources of metals derived from mining, petrochemical industry, printing, electronic industry and municipal waste are ultimately discharged into the marine environment^2^. Seaweeds can rapidly accumulate elevated concentrations of metals, such as Cd and Cu^3,4^. Accordingly, once toxic metals enter aquatic systems where seaweeds grown, they may deposit in human body by intake of these seaweeds finally. Some metals such as Cd, Hg, and Pb can be toxic even at trace levels and biologically essential elements might cause toxic effects at elevated concentrations. Heavy metals accumulate in the fatty tissues and internal organs of human body, which may affect the central nervous system. Noticeably, arsenic, metalloid element, presents different forms which show different toxicities. Inorganic arsenic shows much more toxicity than organic arsenic, which is genotoxic and is a known human carcinogen associated especially with liver, bladder, lung and skin cancer^5^. Except Hijiki (*Hizikia fusiforme*), most of seaweeds naturally accumulate arsenic as virtually nontoxic arsenosugars^6^.

For most countries, there is no regulation on the maximum levels of heavy metals in seaweeds. French legislation has set 0.5 mg/kg as a maximum level for Cd in dried seaweed. Unexpectedly, for Asia, the largest area of seaweed yield, there are no related limits of heavy metals, though element analysis in seaweeds from China, Japan and Korea has been conducted^7,8^.

With rapid urbanization and industrialization, coastal areas of China are now facing serious environmental problems, such as heavy metal contamination^2,9,10^. Some studies have pointed out the heavy metal pollution in soil and sediments along Zhejiang coast^11,12^. Regular surveys on toxic metals in seaweeds and estimation the health risk should be considered to protect consumers. However, to our knowledge, there were little related reports from China.

**Figure 1:**
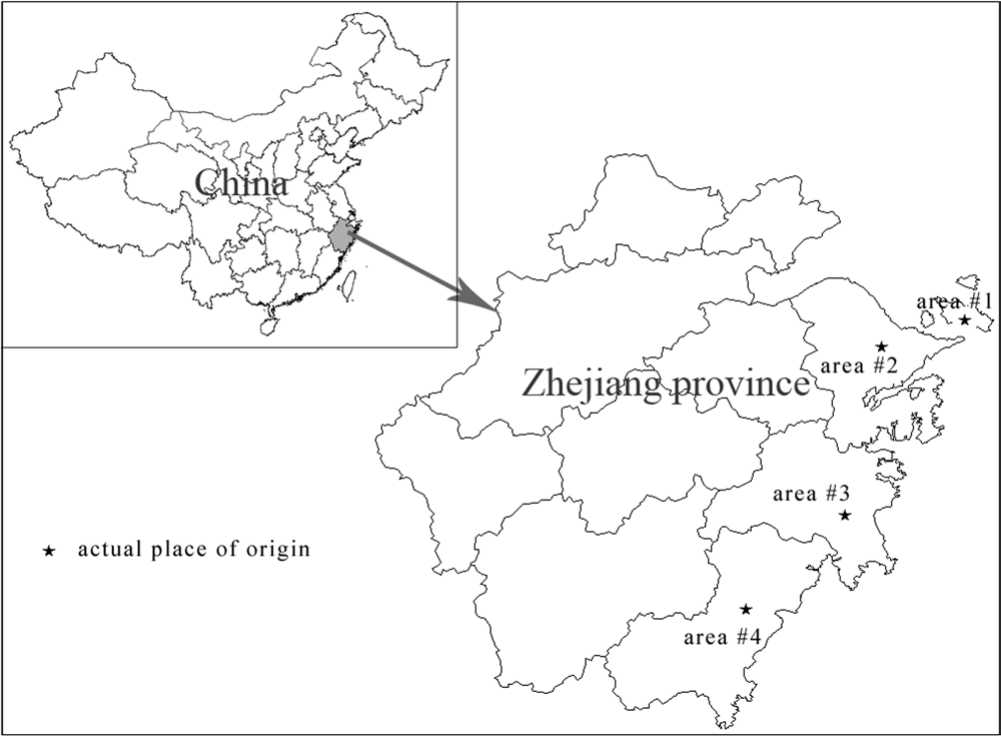
The simple map of the research areas (drawn by software of MapGIS K9 SP2).

This study aimed to analyze metals and metalloids in seaweeds and evaluate the health risk to local inhabitants. Total 295 seaweed samples were purchased from Zhejiang which is a fast developing region in southeast of China (Fig. 1).

## Results and Discussion

### Validation of analysis method

The results for element levels of certified reference materials (CRMs) (GBW10023 and GBW08517) (*n* = 6) are summarized in Table 1. Quantitative results (within 10% of the certified value) were obtained for all elements in each CRM. Mean recoveries were ranged between 82.6–99.4%. For the quality control experiment, reference materials were analyzed along with samples. The relative standard deviations of reference materials between tested value and labeled were less than 10% (except for Hg, it is 15%), so we considered these data of samples be acceptable.

**Table 1:** Results of elemental levels in CRMs.

Elements	GBW10023 Red seaweed			GBW08517 Brown seaweed		
	Certified^a^ mg/kg	Measured^a^ mg/kg	Recovery^b^ (%)	Certified^a^ mg/kg	Measured^a^ mg/kg	Recovery^b^ (%)
Cd	0.57 ± 0.05^c^	0.52 ± 0.04^c^	89.6	1.14 ± 0.11	1.02 ± 0.08	89.5
Al	4.9 ± 0.8^d^	4.8 ± 0.9^d^	98.0	—	—	—
Mn	68 ± 3	66 ± 4	97.1	—	—	—
Ni	2.25 ± 0.18	2.18 ± 0.22	96.9	0.71 ± 0.09	0.68 ± 0.12	95.8
Pb	2.05 ± 0.15	1.95 ± 0.11	95.1	1.41 ± 0.12	1.37 ± 0.14	97.2
Cu	12.2 ± 1.1	12.1 ± 1.8	99.2	5.01 ± 0.32	4.98 ± 0.43	99.4
Se	0.124 ± 0.014	0.109 ± 0.01	87.9	0.062 ± 0.009	0.06 ± 0.007	96.8
Cr	2.4 ± 0.4	2.3 ± 0.5	95.8	0.63 ± 0.08	0.59 ± 0.09	93.7
Hg	0.016 ± 0.004	0.014 ± 0.005	87.5	0.23 ± 0.08	0.19 ± 0.05	82.6
As	27 ± 6	25 ± 5	92.6	13.9 ± 2.4	12.8 ± 2.8	92.1

^a^Mean ± standard deviation.

^b^The percentage of mean measure values to certified.

^c^µg/kg.

^d^g/kg.

### Levels of metals and metalloids in seaweeds

As shown in Table 2, the levels of Cd, Cu, Mn and Ni in red seaweeds were significantly higher than those in brown seaweeds (*P* < 0.01). Levels of Hg and Se in red seaweeds were significantly lower than in brown seaweeds (*P* < 0.05). Similarly, Rubio *et al*.^13^ pointed out red seaweeds showed a tendency to have higher concentrations of Cd than brown seaweed. For all seaweed samples, elements with mean values can be sequenced in descending order, Al > Mn > As > Cu > Cr > Ni > Cd > Se > Pb > Hg. Our results were close to report of Desideri *et al*.^14^ with mean levels of Al (736 mg/kg), As (19.3 mg/kg) and Cd (1.8 mg/kg) in Italy. But, Al (553.8 mg/kg) and Pb (0.595 mg/kg) in this study were higher than studies in Spain with Al (<50 mg/kg) and Pb (<0.2 mg/kg)^13,15^. Cu (6.135 mg/kg) was similar with in Chile with Cu (7.46 mg/kg)^16^. Pb concentration (0.595 mg/kg) was lower than in India and Algeria^17,18^. The difference of these elements content in countries may be caused by the diverse growing environment, as well as the seaweed species. Previous study revealed that eight genera of seaweed had significant differences (*P* < 0.05) in levels of most of trace elements^19^. Furthermore, the data of each metal and metalloid level have large standard deviations. It may be caused by the samples collected from different origins.

**Table 2:** The levels of analyzed elements in red and brown seaweeds.

Elements	Red seaweed (*n* = 142)		Brown seaweed (*n* = 153)		Combine (total *n* = 295)		
	Mean ± SD (mg/kg)	Range (mg/kg)	Mean ± SD (mg/kg)	Range (mg/kg)	Mean ± SD (mg/kg)	P97.5 (mg/kg)	Range (mg/kg)
Al	597.6 ± 594.23	0.353‒3904	597.65 + 655.65	0.173‒4505	553.8 ± 626.4	2376.8	0.173‒4505
As	22.05 ± 11.28	0.185‒41.4	23.01 ± 15.67	0.442‒71	22.61 ± 14.0	52.1	0.185‒71
Cd**	2.225 ± 1.230	0.045‒6.4	0.245 ± 0.286	0.002‒2.09	1.077 ± 1.309	4.615	0.002‒6.4
Cr	2.545 ± 4.080	0.1‒16.6	2.465 ± 4.277	0.08‒35.7	2.498 ± 4.189	15.4	0.089‒35.7
Cu**	11.049 ± 6.277	0.54‒27.1	2.330 ± 5.468	<LOD‒39.2	6.135 ± 7.255	22.85	<LOD‒39.2
Hg**	0.010 ± 0.017	<LOD‒0.132	0.055 ± 0.0619	<LOD‒0.391	0.0370 ± 0054	0.167	<LOD‒0.391
Mn**	43.97 ± 52.30	1.13‒407	18.95 ± 13.72	0.404‒63.8	29.87 ± 38.01	75.3	0.404‒407
Ni**	1.642 ± 1.211	0.32‒7.56	1.123 ± 1.219	<LOD ‒8.82	1.338 ± 1.240	5.475	<LOD‒8.82
Pb	0.655 ± 0.474	<LOD‒3.1	0.539 ± 0.630	<LOD ‒6.96	0.595 ± 0.562	1.872	<LOD‒6.96
Se*	0.466 ± 0.514	<LOD‒2.1	1.053 ± 2.157	0.1‒12	0.797 ± 1.676	7.135	<LOD‒12

“*” and “**” mean that theelemental content between red seaweed and brown seaweed is significantly different at the level of *P* < 0.05 and *P* < 0.01, respectively.

The present study only tested the levels of total arsenic. Usually organic arsenics, for example arsenosugars, have less toxicity than inorganic arsenics. Seaweeds contain arsenic primarily in the form of arsenosugars, which can be metabolized to a wide range of arsenic compounds. The metabolite, such as thio-dimethyl arsenic (thio-DMA) has cytotoxicity in bladder and lung cells^6^. A recent study of Taylor *et al*. investigated the metabolites of arsenosugars in human urine and found only low level of thio-DMA^6^.

The Pearson’s correlation analysis reveals the pairwise associations for a set of variables and determines their direction and strength. The result was showed in scatter plot matrix for the correlation (Table 3). It was observed that no strong correlation (*r* > 0.6) between the contents of studied elements. Weak correlation was found for Ni-Cr (*r = *0.59, *P* < 0.01), Mn-Cu (*r* = 0.29, *P* < 0.01) and Mn-Pb (*r* = 0.22, *P* < 0.01). The content of Al-Cd, Cd-Cr, Cr-Cu, Hg-Mn, Ni-Pb and Al-Se showed very weak correlation (0.1 < *r* < 0.2, *P* < 0.05).

**Table 3:** The correlation of each pair of elements.

Variable	by Variable	Correlation (r)	Significant Probability (*P*)
As	Al	−0.0128	0.7618
Cd	Al	−0.0419	0.4375
Cd	As	0.0671	0.2800
Cr	Al	0.1314	0.0391*
Cr	As	0.0096	0.8769
Cr	Cd	−0.1235	0.0462*
Cu	Al	−0.0206	0.7785
Cu	As	−0.0320	0.6833
Cu	Cd	0.1380	0.0771
Cu	Cr	−0.1920	0.0135*
Hg	Al	−0.0397	0.5867
Hg	As	0.1105	0.0801
Hg	Cd	−0.0883	0.1622
Hg	Cr	0.1125	0.0745
Hg	Cu	−0.0658	0.4009
Mn	Al	0.0182	0.7995
Mn	As	−0.0364	0.6428
Mn	Cd	0.0877	0.2624
Mn	Cr	−0.0761	0.3315
Mn	Cu	0.2906	0.0002*
Mn	Hg	0.1968	0.0113*
Ni	Al	0.0462	0.4432
Ni	As	−0.0094	0.8804
Ni	Cd	0.0206	0.7401
Ni	Cr	0.5954	<0.0001*
Ni	Cu	−0.0075	0.9242
Ni	Hg	−0.0006	0.9930
Ni	Mn	−0.0297	0.7050
Pb	Al	0.0224	0.6615
Pb	As	0.0229	0.7133
Pb	Cd	−0.0029	0.9621
Pb	Cr	0.0983	0.1132
Pb	Cu	−0.0270	0.7306
Pb	Hg	0.0731	0.2477
Pb	Mn	0.2218	0.0042*
Pb	Ni	0.1423	0.0214*
Se	Al	−0.1521	0.0362*
Se	As	0.0645	0.4104
Se	Cd	−0.1296	0.0970
Se	Cr	−0.0776	0.3216
Se	Cu	−0.1245	0.1112
Se	Hg	−0.0629	0.4220
Se	Mn	−0.0623	0.4268
Se	Ni	−0.1393	0.0744
Se	Pb	−0.0723	0.3560

### Seaweeds from different geographical origins

The levels of elements in brown and red seaweeds from different origin were showed in Table 4. The results for combination of above brown and red seaweeds were also calculated. For brown seaweeds, levels of Al, Cd, Cu, Hg, Mn and Se existed significant differences in different origin areas (*P* < 0.05). For red seaweeds, elements with obvious difference in areas were Al, As, Cu, Mn and Pb. When the data of red and brown seaweeds were combined, the Al, Hg and Pb showed significant difference in terms of origin areas.

**Table 4:** The levels of elements in seaweed from different origins.

Seaweeds	Area	Levels of elements (mean ± SD) mg/kg									
		Al	As	Cd	Cr	Cu	Hg	Mn	Ni	Pb	Se
Brown	1	810.3 ± 65.8^a^	25.78 ± 7.06	0.217 ± 0.100^b^	2.7 ± 0.964	1.255 ± 0.370^abc^	0.087 ± 0.063^a^	21.13 ± 8.60^ab^	1.028 ± 0.600	0.741 ± 0.282	0.329 ± 0.104^a^
	2	1072 ± 207.4^b^	30.5 ± 13.4	0.158 ± 0.086^b^	1.774 ± 0.773	1.413 ± 0.720^ab^	0.041 ± 0.017^b^	27.13 ± 13.8^a^	1.031 ± 0.549	0.532 ± 0.422	0.583 ± 0.286^a^
	3	729.6 ± 264.1^a^	27.54 ± 7.70	0.480 ± 0.134^a^	3.061 ± 1.982	1.998 ± 0.418^a^	0.059 ± 0.019^ab^	23.93 ± 9.62^ab^	1.023 ± 0.527	0.536 ± 0.187	0.217 ± 0.093^a^
	4	394.4 ± 55.6^c^	25.36 ± 12.74	0.117 ± 0.085^b^	2.977 ± 1.062	1.099 ± 0.782^bc^	0.029 ± 0.030^b^	11.30 ± 9.38^bc^	1.392 ± 1.272	0.543 ± 0.298	1.533 ± 1.299^b^
Red	1	260.7 ± 60.4^a^	12.44 ± 4.76^B^	1.942 ± 0.983	5.493 ± 1.537	5.161 ± 3.280^B^	0.008 ± 0.005	17.00 ± 4.88^B^	1.156 ± 1.022	0.764 ± 0.202^AB^	0.073 ± 0.132
	2	523.7 ± 78.5^b^	27.48 ± 9.04 ^A^	2.426 ± 1.588	2.419 ± 1.356	13.418 ± 6.077^AB^	0.017 ± 0.029	44.44 ± 16.47 ^A^	1.646 ± 0.847	0.670 ± 0.345^B^	0.226 ± 0.086
	3	621.9 ± 188.9^b^	29.62 ± 4.55^AB^	3.076 ± 1.306	6.846 ± 1.892	21.940 ± 2.907 ^A^	0.009 ± 0.003	46.36 ± 15.50 ^A^	2.138 ± 1.256	1.186 ± 0.727 ^A^	0.127 ± 0.033
	4	522.0 ± 102.3^b^	22.74 ± 10.30^AB^	2.227 ± 1.220	2.543 ± 1.298	10.524 ± 6.955^B^	0.008 ± 0.008	46.56 ± 25.6 ^A^	1.952 ± 1.790	0.603 ± 0.432^B^	0.548 ± 0.537
Combine	1	590.4 ± 113.9^a^	20.86 ± 9.04	3.729 ± 1.209	0.853 ± 0.314	2.694 ± 1.589	0.062 ± 0.064^×^	19.61 ± 7.57	1.075 ± 0.756	0.750 ± 0.248^xy^	0.235 ± 0.169^×^
	2	715.6 ± 126.1^b^	29.06 ± 11.41	2.084 ± 1.006	1.292 ± 0.867	7.129 ± 2.356	0.030 ± 0.026^yz^	35.37 ± 17.21	1.327 ± 0.762	0.618 ± 0.376^xy^	0.413 ± 0.279^×^
	3	663.3 ± 129.7^b^	28.23 ± 6.71	4.323 ± 1.025	1.345 ± 0.495	8.645 ± 2.705	0.042 ± 0.029^xy^	31.41 ± 15.75	1.395 ± 0.962	0.926 ± 0.654^×^	0.187 ± 0.088^×^
	4	481.9 ± 96.2^a^	23.67 ± 11.16	2.697 ± 1.247	1.509 ± 0.660	6.366 ± 2.663	0.016 ± 0.021^z^	31.01 ± 42.42	1.754 ± 1.634	0.583 ± 0.391^y^	0.982 ± 1.058^y^

a,b,c,A,B,C,x,y and z mean data with upper letters where do not share one same alphabet shows that there are significant differences among areas by One-way ANOVA and Tukey HSD multiple comparisons (*P*<0.05).

**Figure 2:**
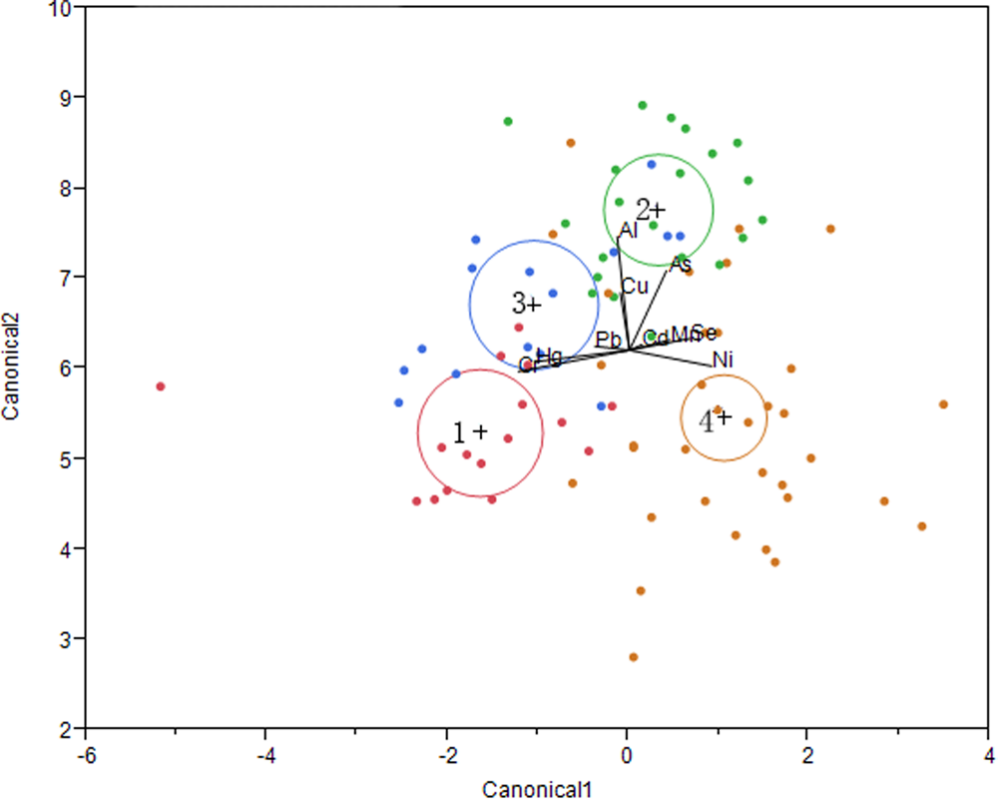
Canonical plot of linear discriminant analysis on the seaweeds from different origins.

Investigators such as Larrea-Marı’n *et al*.^20^ and Akcali *et al*.^21^ have emphasized the importance of the culture region to changes of elemental levels in seaweeds. To identify the geographical origin of seaweeds, we performed linear discriminant analysis (LDA) with stepwise procedure. LDA can maximize the variance between groups and minimize the variance within the group by creating new variables Canonical plot of linear discriminant analysis were showed in Fig. 2. Samples from different origin focus in different labeled circles. The size of the circle corresponds to a 95% confidence limit for the mean. Groups that are significantly different tend to have non-intersecting circles. “ + ” sign is a marker for the center of the circle. On the whole, 67.4% seaweed samples were correctly classified. The origin area 1 had the best performance with only 6.3% samples misclassified. The discrimination functions were listed as bellow. These functions can be used for predicting the origins of unknown seaweeds. So when we want to predict the place of a sample, we just calculate the value by the function equation and observe the distribution in circles.

Canon 1 (*x*) = −20.87Hg + 0.027As − 0.222Cr + 0.650Se − 0.586Pb − 0.009Cu − 0.001Al + 0.011Mn + 0.063 Cd + 0.554Ni.

Canon 2 (*y*) = −2.54Hg + 0.060As − 0.042Cr + 0.126Se + 0.067Pb + 0.059Cu + 0.007Al + 0.003Mn + 0.028 Cd − 0.107Ni.

Canon 3 (*y*) = −4.78Hg + 0.034As − 0.062Cr + 0.154Se + 1.978Pb + 0.088Cu− 0.003Al − 0.004Mn − 0.076 Cd + 0.311Ni.

### Exposure assessment

As shown in Table 5, the exposure doses of elements were calculated at the mean and P97.5 level. For As, we adopt 5% of value of total As to calculated the exposure, because in seaweed about 90% total As were organic arsenic, especial arsenosugars which shown very little toxicity^5^. The targeted hazard quotient (THQ) for single element was less than 1. Hazard index (HI) was 0.49 for mean exposure and 1.68 for high exposure. It indicates that there is low health risk for toxic elements by intake of seaweeds. However, in terms of the high exposure level (P97.5), they may be the potential adverse effect on human health. Several studies also claimed that total elemental intake does not appear to pose any threat to the consumers in Italy, South Korea and Spain^13,14,22^, though different safety reference values, such recommended reference dose (RfD) from US EPA and provisional tolerable weekly intake (PTWI) from the Joint FAO/WHO Expert Committee were used for assessment.

**Table 5:** Estimated exposures for the general population in seaweeds from Zhejiang province.

Element	RfD^a^ μg/kg bw/day	Exposure Dose μg/kg bw/day		THQ	
		Mean^b^	P97.5^c^	Mean	P97.5
Al	1000	41.1	176.6	0.04	0.18
Cd	1	0.08	0.34	0.08	0.34
Cr	3	0.19	1.14	0.06	0.38
Cu	40	0.46	1.70	0.01	0.04
Hg	0.3	0.003	0.012	0.01	0.04
Mn	—	2.22	5.59	—	—
Ni	20	0.10	0.41	0.00	0.02
Pb	3.6	0.04	0.14	0.01	0.04
Se	—	0.06	0.53	—	—
				HI^d^: 0.22	HI: 1.05

^a^RfD, recommended reference dose for chemicals provided by US EPA.

^b^The mean levels of elements were used.

^c^The P97.5 levels of elements were used.

^d^HI, hazard index calculated by sum of THQ; Mn and Se were not included.

For calculating exposure dose, we adopted 5.2 g/capita/day as the consumption of seaweeds in China, which is higher than in Japan for 4 g/adult/day^23^ and lower than in South Korea for 8.5 g/adult/day^22^. In addition, it should be noted that the risk assessment on arsenic was not performed because the arsenic species were not analyzed here. The toxicity of organic arsenic is lower than inorganic species^24^. The study about organic arsenic exposure including arsenolipids and arsenosugars was recently reported by Taylor *et al*.^6^.

### Uncertainty of the risk assessment

It should be noted that HI used for risk assessment was based on concentration/dose addition of different metals and metalloid. The addition of each value is a non-interactive process, which means that metals and metalloid in the mixture do not affect their toxicity. Actually, multiple metals in human body may have the interactive effect. Furthermore, loss of elements caused by the cooking and absorption ratio of human intestine was not considered here. So, the above factors may lead to the uncertainty of the estimates.

Usually, the average level of targeted chemicals was adopted for dietary exposure assess. For avoiding the underestimation of the intake, we used both mean and P97.5 value of element concentration for the exposure assessment.

## Conclusion

The present study revealed the different levels of Al, As, Cd, Cr, Cu, Hg, Mn, Ni, Pb and Se in dried seaweeds from southeastern China (Zhejiang province). It indicates that element concentration changes with different species of seaweeds and origin areas. For example, the levels of Cd, Cu, Mn and Ni in red seaweeds (*Phorphyra*) were significantly higher than those in brown seaweeds (*Laminaria* and *Undaria*) (*P* < 0.01). The estimate of health risk showed that there was low health risk for potential toxic elements by intake of these seaweeds. However, in terms of the hazard of some metals to human body, continuous surveillance in edible seaweeds is necessary for health protection to consumers^25^. Furthermore, for Asian countries, regulations on maximal concentration of heavy metals in seaweed products should be set up.

## Materials and Methods

### Sampling

Dried seaweeds were collected in Zhejiang, China, where the latitudes range from 27°09′ to 31°11′N, and the longitudes from 118°02′ to 122°57′E from April to November, 2016. Four places of seaweed origin in Zhejiang coast were marked as 1, 2, 3 and 4 in Fig. 1. The simple map (Fig. 1.) was drawn by software of MapGIS K9 SP2 free trial edition (Zondy Cyber Comp., China). The total 295 seaweed samples included 142 red seaweeds (*Phorphyra*) and 153 brown seaweeds (*Laminaria* and *Undaria*). All samples were presented at the dehydrated (dried) state, and they were stored at 4 °C and immediately analyzed within 24 h.

### Laboratory analysis

The levels of Al, As, Cd, Cr, Cu, Hg, Mn, Ni, Pb and Se in dried seaweed were analyzed by microwave digestion and ICP-OES based on our previous report^26^. Briefly, the samples (0.5~2 g) and 6 ml nitric acid were transferred to Teflon-PFA vessels. Samples were digested at180 °C for 30 min by a MARS Xpress Microwave Digestion System (MARS 6, CEM Corporation, Mathews, NC, USA).Afterward, the digested solutions were evaporated to 0.5 ml with an electro-thermal plate and finally diluted to 5 or 10 ml with de-ion water for instrumental analysis. The solution was analysis by ICP-OES (Agilent 720ES, Agilent technologies Inc., USA). Limits of detection (LODs) were defined as 3 times the standard deviation of 10 runs of blank measurements. LODs of Al, As, Cd, Cr, Cu, Hg, Mn, Ni, Pb and Se were 0.015, 0.012, 0.009, 0.008, 0.009, 0.005, 0.007, 0.008, 0.010 and 0.045 mg/kg respectively.

### Quality control

We selected certified reference materials (CRMs) as the quality control (QC) samples. Two CRMs, GBW10023 red seaweed (*Phorphyra*) and GBW08517 brown seaweed (*Laminaria*), were purchased from National Research Center for Certified Reference Materials, China (NRCCRM) (Table 1).

### Consumption data of dried seaweed

There are no related data of seaweed in Zhejiang provincial dietary consumption survey. So, we calculated consumption data based on the production quantity in mainland of China. The intake value of 25.87 g/capita/day seaweed was obtained in website of Food and Agriculture Organization of the United Nations (FAO) (derived from FAO DATA STATS, http://www.fao.org/fishery/topic/16140/en). Considering water content of fresh seaweeds, like vegetables, is 70–90%^27,28^, we used 5.2 g/capita/day (20% of original value) as the consumption data for dried seaweed.

### Health risk assessment

The targeted hazard quotient (THQ) and hazard index (HI) were used toestimate health risk according to US EPA’s IRIS database^29^. We used the mean and 97.5th percentile of obtained elements concentration to represent the consumers with average and high exposure, respectively^30^. The sum of all THQs for each element is referred to as the HI. The formulas are as follows:

1 $$\mathrm{Exposure}\,\mathrm{Dose}=\frac{Ci\times Di\times Ed}{Bw\times At}$$

2 $$\mathrm{Tageted}\,\mathrm{Hazard}\,\mathrm{Quotient}\,({\rm{THQ}})=\frac{\mathrm{Exposure}\,\mathrm{Dose}}{RfD}$$

3 $$\mathrm{Hazard}\,\mathrm{Index}\,({\rm{HI}})=\sum _{k=1}^{n=k}\mathrm{Targeted}\,\mathrm{Hazard}\,\mathrm{Quotient}$$

Ci is the average or P97.5 concentration of the element in the seaweeds (mg/kg); Di is the daily intake of seaweeds (5.2 g/capita/day); Ed is the average exposure duration (e.g., 70 years); Bw is the average weight (e.g., 70 kg); At is the average lifetime (e.g., 70 years). RfD is the recommended reference dose (RfD); According to US EPA guidelines for assessing conservative risk, HI were calculated by sum of the THQ. When HI < 1, no health risk is expected to occur; If HI ≥ 1, there is moderate or high risk for adverse human effects.

### Statistics

One-way ANOVA and Tukey HSD for comparing means was used for observe differences between different seaweeds or different origins. Pearson correlation was used to analyze linear relationships among different elemental concentrations. Linear discriminant analysis (LDA) was used for identify seaweeds from different growing regions by quantified elements. All these statistical methods were performed on software of JMP 10.00 free-try edition (SAS Institute Inc.). In addition, data used for exposure estimates were according to the recommendation of the report Reliable Evaluation of Low-Level Contaminations of Food issued by WHO^31^. Thus, a value of ^1^/_2_ LOD was assigned to all results below the LOD, where the proportion of <LOD results is not > 60%.
